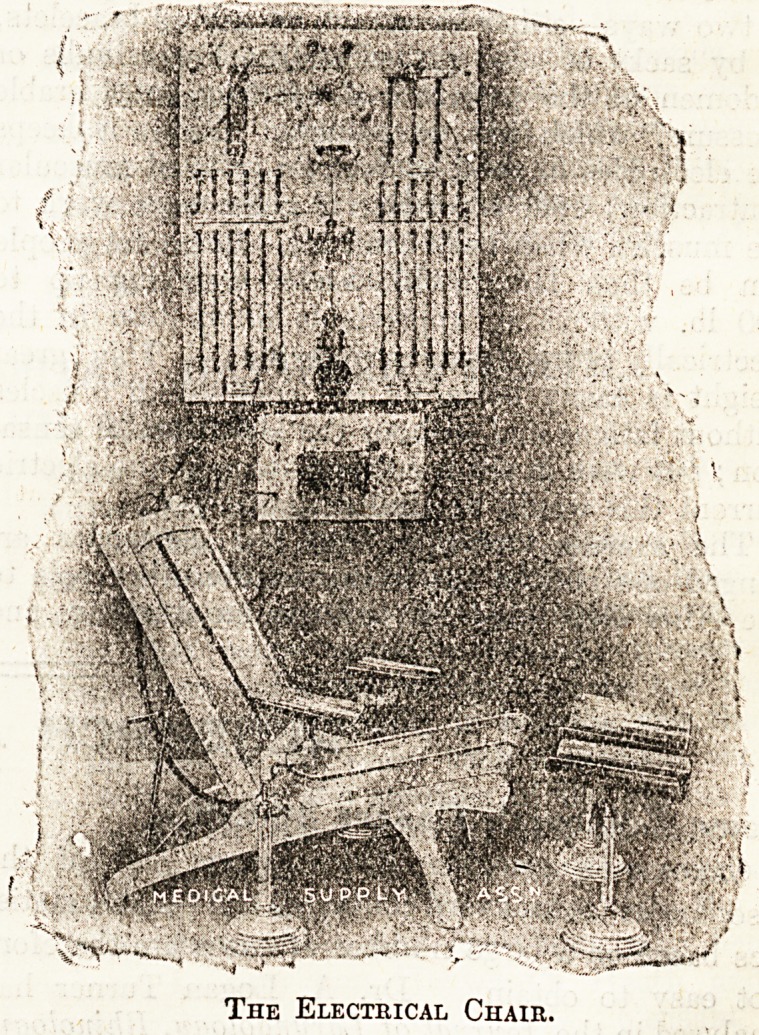# Electricity and Obesity: Treatment by Electrically Excited Exercise

**Published:** 1914-03-28

**Authors:** 


### ELECTRICITY AND OBESITY.

### Treatment by Electrically Excited Exercise.

Electricity has been successfully used in the treatment of obesity by the method of Bergonié for some fifteen years in France, and was introduced into this country in 1911. The great advantage claimed is that from the beginning of the treatment the patients notice an improvement in general health; a feeling of *bien-être* and lightness to which they are unaccustomed.

In some of the diet systems too often the result is that, although weight may be lost, the patient looks much older, whereas after a course of Bergonié the patient is stated to look much younger. Not only is the patient improved in appearance, but the breathing, if difficult before, has become easier, and the heart is strengthened; in fact, as has been pointed out by Dr. Hampson before the Royal Society of Medicine, this treatment is of extreme value in cases of cardiac mischief, especially those with dilatation or feeble musculature.

The principle upon which the treatment is based is merely to make the muscles contract and relax by electrical stimulation. Thus exercise is obtained without fatigue, a desideratum which has long been sought and is of great therapeutic value. The current is derived from what may be termed an induction coil, or a coarse wire faradic whose coefficient of transformation is two or three (*i.e.*, four volts in the primary and eight or twelve in the secondary); below the coil are the condensers. The current is rhythmically interrupted and reversed by means of a special noiseless and springless metronome about a hundred times a minute, the aim being to obtain muscular contractions of maximum strength, with a minimum of—one cannot say pain, for there is no pain with this treatment—sensation.

In order to accomplish this object there must be a perfect regularity of the waves induced by the make and break of this current; they must be regular, equal, and synchronous. For, while a muscle contraction is agreeable and painless if produced by such waves, it is equally disagreeable and even insupportable if these waves are unequal in form or frequency. One of the requisite conditions to insure this regularity is that the interrupter must act in a perfectly even manner, and the old-fashioned simple ribbon interrupter, weighted at its extremity with a piece of iron, seems to give the greatest satisfaction. The length can be varied so as to give a musical sound of as pure a tone as possible; a little experience soon teaches this. The tone is also modified by the amount of current allowed to flow through the coil. The binding posts should be fastened with screw nuts into the board, and the screw bearing the platinum point should not work too easily. When working no spark should be visible, even if the room be darkened, and this is a condition most difficult to obtain, but indispensable in order to secure painless energetic muscular contractions. A condenser of suitable capacity, properly adjusted, is of primary importance; one which could be varied would be an ideal adjunct to this apparatus. In practice, the main commercial direct current is led through a resistance bank of lamps and through a wire rheostat, a volt meter and an ampere meter are put in circuit, and the current led to the coil. It is varied by means of the wire rheostat until there is no sparking in the interrupter, and the requisite musical note is heard.

The quantity of current is quite considerable, and the average reading in the primary is 2.5 amperes at 24 volts. Thus it will be seen that the apparatus, apart from its other modifications, is something quite different from the faradic coil of former times.

The interrupter should work at a frequency of about thirty times per second. The faradic current on its way to the patient then undergoes longer interruptions with reversals. The rate which produces the best results and is most comfortable to the patient is when the latter interrupter is set at a rate of 100 to 120 times per minute.

The Electrical Chair.

As the object aimed at is the simultaneous stimulation of the greatest number of muscles of the body possible, the electrodes should be of as large surfaces as possible. They are of two kinds —stationary and movable. The stationary electrodes used are always the same, irrespective of the size and weight of the patient; they take the form of a semi-reclining chair, of which they constitute the seat and back, two forming the seat and two forming the back, being separated each from the other by a small space. They are made of metal, and connected with the points of exit on the wall-plate. Each electrode is covered with a towel wrung out of warm water, and the patient, clothed only in a light dressing-gown, seats himself on the chair. The movable electrodes are semi-cylindrical pieces of metal, varying in surface, form, curve, etc., and are placed with the intervening warm, wet towel on the thigh, under the calf (resting on a special support), on the abdomen, and on the arms or breasts—twelve electrodes in all. The total surface covered by these electrodes is very large, and may, in some very fat people, be even 10,000 square centimetres. The resistance which the body offers naturally varies considerably, but Professor Bergonié has measured it, with all the electrodes well applied and well wetted, at less than 200 ohms.

The movable electrodes may be held in position in two ways—either by means of rubber bracelets, or by sacks of sand placed on the lower limbs or abdomen of the patient and exerting considerable pressure, which has advantages. First, it keeps the electrodes in good contact, in spite of muscular contraction; and, secondly, it gives more work to the muscles when they contract. Some fat people can be thus laden with sacks weighing up to 200 lb. without an appreciable diminution in the electrically stimulated movements. This great weight is easily borne by the contracting muscles without fatigue and without the least painful sensation; but were it not for the passage of the electric current this would be almost impossible.

The average current, with the electrodes arranged one pole to the back and the other pole to the other electrodes, the metronome working, and the interrupter well regulated, produces good contractions in a man of ordinary muscles; 25 to 30 milliamperes are sufficient. But in very fat women with muscles poorly developed 70 to 80 milliamperes may be registered on the meter without the sensation being in any way painful. The treatment should never be tiresome, but if at first it be too violent or of too long duration there will be some muscular stiffness. As a rule, it is best to begin with a treatment of twenty minutes, and increase by five minutes daily until forty or forty-five minutes is reached.

Patients undergoing this treatment should be under continuous medical supervision. In a patient in whom great metabolic changes are daily taking place it is obvious that the medical adviser should keep a watch upon the patient, listening to the heart, examining the urine—especially for albumen and acetone—watching for and guarding against constipation, and in many other ways which will suggest themselves during the course of the treatment; with this careful supervision no harm can possibly come, but benefit will certainly ensue from a prolonged course of this electrically excited exercise.

The diet during the treatment must vary with the individual, and with what he has been in the habit of eating and drinking. *Prima facie*, with so much animal combustion going on it should merely consist of fruit, vegetables, and salad; in fact, it may be said that by the aid of this current one eats one's self, and a little salad with it. As a rule, patients take very kindly to the treatment, and after the first few days the improvement in the general appearance is such that they are often more anxious to make the diet more strict than to offer objections to the limitations imposed by the physician.

## Figures and Tables

**Figure f1:**